# Assessing trauma care health systems in low- and middle-income countries, a protocol for a systematic literature review and narrative synthesis

**DOI:** 10.1186/s13643-019-1075-8

**Published:** 2019-07-02

**Authors:** John Whitaker, Max Denning, Nollaig O’Donohoe, Dan Poenaru, Elena Guadagno, Andy Leather, Justine Davies

**Affiliations:** 1 King’s Centre for Global Health and Health Partnerships, School of Population Health & Environmental Sciences, Faculty of Life Sciences and Medicine, King’s College London, Room 2.13, Global Health Offices, Weston Education Centre, Cutcombe Road, London, SE5 9RJ UK; 2 Department of Surgery and Cancer, Imperial College London, London, UK; 3 Lewisham and Greenwich NHS Trust Hospital, London, UK; 4 Division of Pediatric General and Thoracic Surgery, McGill University Health Centre, Montreal, Canada; 5 Centre for Applied Health Research, University of Birmingham, Birmingham, UK; 6 Medical Research Council/Wits University Rural Public Health and Health Transitions Research Unit, Faculty of Health Sciences, School of Public Health, University of the Witwatersrand, Johannesburg, South Africa

**Keywords:** Trauma, Injury, Health system, Assessment, Evaluation, LMIC, Low-income country, Middle-income country

## Abstract

**Background:**

Trauma represents a major global health problem projected to increase in importance over the next decade. The majority of deaths occur in low- and middle-income countries (LMICs) where survival rates are lower than their high-income country (HIC) counterparts. Health system level changes in care for injured patients have been attributed to significant improvements in care quality and outcomes in HIC settings. There is a need for further research to assess trauma care health systems in LMICs to inform health system strengthening for the care of the injured. This study aims to conduct a narrative synthesis of a systematic search of the literature on the assessment of trauma care health systems in LMICs in order to inform the further development of trauma care health system assessment.

**Methods:**

The review will include primary quantitative, qualitative or mixed method studies and secondary literature reviews. No restriction will be placed on language or date. Reports and publications identified from the grey literature including from relevant national and international health organisations will be included. Articles will be screened by two independent reviewers with a third reviewer resolving any persisting disagreement. The search will reveal heterogenous studies not suitable for meta-analysis. A narrative synthesis of the identified papers will be conducted to identify key methodological ideas and paradigms used to assess trauma care health systems. The analysis will consider how the differing methodological approaches could be adopted to understand barriers and delays to seeking, reaching and receiving care within a “Three Delays” framework. An iterative approach will be adopted to categorise identified articles, with the results presented as both within and across study analysis.

**Discussion:**

The results of the review will be disseminated through publication in a peer-reviewed academic journal. The study forms part of a PhD project. The results will inform the development of a trauma care health system assessment applicable to LMICs. As this is a review of secondary data, no formal ethical approval is required.

**Systematic review registration:**

PROSPERO CRD42018112990

**Electronic supplementary material:**

The online version of this article (10.1186/s13643-019-1075-8) contains supplementary material, which is available to authorized users.

## Background

### Rationale

Trauma represents a major global health problem with injuries accounting for more deaths than TB, malaria and HIV combined and with 90% of these deaths occurring in low- and middle-income countries (LMICs) [1]. Along with other non-communicable diseases, death from trauma is set to increase with some projecting road traffic collisions to be the third leading cause of death by 2030 [2]. Non-fatal injuries are common, with 1 billion people sustaining an injury in 2013 that warranted health care [3]. There is also considerable global variation in injury-related morbidity. Disability-adjusted life years (DALYs) in children are 9 times greater in sub-Saharan Africa compared to those in high-income Asia Pacific counterparts [3], likely due to both differences not only in preventative measures but also in the injury care available from health systems. Indeed, if the survival rates following injury in LMICs were to be improved to the rates seen in HICs, the estimated one third of annual global trauma deaths could be avoided [4].

Considering and developing the whole system of trauma care from point of injury to rehabilitation services has resulted in significant improvements in trauma care in high-income country (HIC) settings. Such improvements were particularly amongst the most severely injured [5–7]. Although prevention is rightly a key focus on reducing the global burden of injuries, better trauma care through system improvement could lead to major reductions in global trauma associated mortality and has been strongly advocated [4, 8]. Furthermore, trauma care has been considered a tracer condition that can be useful in assessing wider emergency health system performance [9, 10]. The World Health Organization (WHO) advocates that promoting essential trauma care will concurrently promote wider health care system improvements beneficial for other urgent surgical and non-surgical emergencies [11]. The Lancet Global Health Commission on High Quality Health Systems has highlighted the disparity between global burden of injuries and the lack of available data on care quality provided by health systems. Better assessment of such care is one of the commission’s stated research priorities [10].

The WHO defines health systems as the “organisations, people and actions whose primary intent is to promote, restore or maintain health” [12]. Health systems have been described as complex adaptive systems that may respond in non-linear, unpredictable ways to interventions [13]. Health systems consist of an intricate web of relationships between the component parts, embedded within social institutions with human behaviours influencing performance and function [14]. Understanding existing health systems, through their assessment, is important to inform impactful health system improvement efforts [15]. Many different frameworks for describing and understanding health systems exist. Such frameworks have their origins in differing paradigms of understanding and sociopolitical backgrounds [16]. Whilst a universal framework for understanding such complex systems may therefore not exist, choosing a particular health system framework of understanding should be done to fit a purpose [16]. One particular framework that has had success in improving understanding and outcomes in maternal mortality is the “Three Delays” model. It was developed to help evaluate the delays to care driving adverse outcome in maternal mortality in LMICs [17]. The framework has been widely adopted in the field of maternal, neonatal and child health in an attempt to evaluate and drive improvements in care [18–22]. It has also been proposed as a framework through which to evaluate emergency healthcare in LMICs including trauma [23]. The Three Delays framework considers the barriers that result in delays in seeking care (delay 1), reaching care (delay 2) and receiving appropriate care (delay 3) [17].

In order to inform the development of future trauma care health system assessment, we will undertake a review of the existing literature on assessing trauma care health systems. Applying the Three Delays framework of analysis to this literature review will allow future development of a health system assessment strategy based on this approach.

### Objective

The objective is to conduct a narrative synthesis of a systematic search of the literature on the assessment of trauma care health systems through a Three Delays model framework of understanding, in order to inform the development of trauma care health system assessment.

## Methods

The 2015 guidelines for Preferred Reporting Items for Systematic Review and Meta-Analysis Protocols (PRISMA-P) have been followed in the design of this protocol (Additional file 1). Any amendments to the protocol, although not anticipated, will be reported when publishing the results. This literature review protocol has been registered with PROSPERO reference number CRD42018112990. It is anticipated the review will be complete by Dec 2019. The study is part of a PhD project, supported by King’s Centre for Global Health and Health Partnerships, Royal College of Surgeons of England and the UK Defence Medical Services.

### Eligibility criteria

We will include primary quantitative, qualitative or mixed method studies and secondary literature reviews. No restriction will be placed on language or date. We will also include reports and publications identified from the grey literature including from relevant national and international health organisations (listed in Appendix 1). The inclusion and exclusion criteria are illustrated in Table 1.

**Table 1:** Tabulated inclusion and exclusion criteria for selecting articles for review

	Include	Exclude
Type of article	Primary quantitative, qualitative or mixed method study Literature review Report or guideline from national or international health organisation	Case reports, academic letter, correspondence or conference proceedings
Type of conditions or care setting	Trauma and injury (used interchangeably) care	Mental health Non-urgent care, primary care, elective care as the main focus of assessment Non-trauma emergency care Non-accidental injury in children Disaster management
Subject of study	Whole health system assessment Assessment of health-seeking behaviour Assessment of community perception of health care access and quality Assessment of health system access Assessment of health system care quality including technical and patient-centred care	Measurement of population health profiles and patterns Research evaluating interventions, diagnostic tests, medicines or technologies
Study setting according to World Bank Income Classification 2018	Includes low- or lower middle- or upper middle-income country	High-income country only

### Search methods

A comprehensive search strategy has been developed to electronically search the following databases from inception: MEDLINE (Ovid), Global Health (Ovid), Embase (Ovid), Web of Science (Clarivate Analytics), Cochrane (Wiley), Global Index Medicus (WHO) and Africa Wide Information (Ebsco). The MEDLINE example can be found in Appendix 2, and the full strategy is available on request. The search strategy uses variations in text words found in the title, abstract or keyword fields, and relevant focused subject headings to retrieve articles combining the concepts of (1) trauma, including disaster planning, mass casualty incidents or emergency care along with (2) various types of assessments, evaluations, benchmarking or tools used to create or improve (3) health system programmes. Three-delay models, rapid assessments and verbal or social autopsies will also be verified. Animal studies will be excluded. Articles will be initially collated in Endnote X8 for de-duplication of results. Screening of abstracts and titles will be done collaboratively using the Rayyan QCRI online open-source web application [24]. There is no agreed gold standard for searching the grey literature; however, a four-stage approach has been advocated for seeking relevant grey literature articles [25]. These complementary approaches are searching of grey literature databases, a customised Google search, targeted websites and consultation with experts [25]. We will search the following grey literature databases using the search terms “trauma” OR “injury” AND “assessment” OR “evaluation” AND “health system” modified according to the database requirements. The grey literature databases to be searched are OpenGrey, WorldCat Dissertations and Theses (OCLC) and New York Academy of Medicine Grey Literature Report and Core. We will use advanced Google searches both with and without limiting the domains to .org, .edu, .int and .gov using combinations of the terms “trauma”, “injury”, “assessment”, “evaluation” and “health system”. The top 50 sites will be screened for relevant articles for each search. We will search the specific websites listed in Appendix 1 using the same search terms. We will also include any additional articles recommended by experts in health system research or trauma care that might inform our review. The reference lists of identified articles will be reviewed for any additional articles of relevance to include.

### Identification of studies

Key term screening within the Rayyan application will be used to remove any clearly identified as animal or cellular studies. Following piloting of the study selection process, two reviewers will independently screen the articles identified, firstly by title and then abstract. Disagreements over eligibility will be discussed in order to achieve consensus. Where disagreement persists, a third reviewer will arbitrate. Full texts of the abstracts will be obtained and assessed for eligibility by two reviewers. Articles not in English will be translated using Google translate where possible. Articles meeting eligibility criteria will proceed to data extraction. Reasons for exclusion will be recorded. Each grey literature database search, Google search, focused website search and expert request will be conducted by one reviewer with a second reviewer confirming eligibility of identified articles. The level of reviewer agreement will be presented in the final report.

### Risk of bias

This review is primarily focused on the methodological approach identified in the article and aims to understand a wide breadth of diverse research approaches used to assess trauma care health systems. The quality of conduct of each specific study and the trustworthiness of results and findings for each article are therefore less important.

### Data extraction

A standardised extraction form will be developed and piloted. Information to be extracted will include author; publication year; study type; study clinical focus; conceptual framework used if applicable; which of the three delays are assessed if applicable; the country location; the methodological approach; and author reported strengths and limitations, including time, pragmatism and cost if reported. Two authors will extract the information independently with a third arbitrating in the case of unresolved disagreement.

### Analysis of results

The search will reveal heterogeneous studies. Meta-analysis of study findings is not a study objective. A narrative synthesis of the identified papers will be conducted to identify key methodologies used to assess trauma care health systems. The analysis will consider how the differing methodological approaches could be adopted to evaluate barriers and delays to care within a Three Delays framework to best understand trauma care health systems. The methods identified will also be assessed for their suitability to be employed in a rapid assessment, specifically their relative resource requirements including time taken to undertake, pragmatism and cost. An iterative approach will be adopted to categorise identified articles, with the results presented as both within and across study analysis.

## Discussion

This narrative synthesis of a systematic search of the literature will summarise the established approaches for assessing trauma care health systems. As part of a PhD project, it will be used to inform the development of a Three Delays model health system assessment of trauma care health systems in LMICs. It is hoped that it will facilitate other health system researchers to develop assessment strategies and facilitate health system strengthening for trauma care. To our knowledge, it is the first attempt to synthesise the literature on health system assessment methods for trauma care.

### Additional file

Additional file 1: PRISMA-P checklist. (DOCX 22 kb)

## Data Availability

Not applicable

## Appendix 1

List of relevant national and international health organisations’ websites to search

- World Health Organization
- World Bank
- USAID
- United Nations Educational Scientific and Cultural Organization
- Medicins Sans Frontiers
- International Committee of the Red Cross
- The International Federation of Red Cross and Red Crescent Societies
- International Federation of Emergency Medicine
- African Federation of Emergency Medicine
- Asian Society of Emergency Medicine
- International Association for Trauma Surgery and Intensive Care
- College of Surgeons of East, Central and Southern Africa
- G4Alliance

## Appendix 2

Search strategy, developed for MEDLINE (Ovid), October 9, 2018.

MEDLINE [Ovid] (October 9, 2018)

Ovid MEDLINE(R) and Epub ahead of print, in-process and other non-indexed citations, Ovid MEDLINE(R) Daily <1946 to present>

**Table:**

1	exp *"Wounds and Injuries"/	685,268
2	exp *Emergency Service, Hospital/	40,504
3	exp *Emergency Medicine/	9475
4	exp *Emergency Treatment/	67,739
5	exp *Emergency Medical Services/	82,657
6	exp *Accidents/sn [Statistics & Numerical Data]	12,878
7	*Traumatology/	2723
8	Traumatology/og, st, sn	889
9	*Disasters/	13,701
10	*Disaster Medicine/	599
11	exp *Terrorism/	8845
12	*Relief Work/	2679
13	*Emergency Shelter/	83
14	*Rescue Work/	1344
15	or/1-14	845,684
16	*"surveys and questionnaires"/	40,830
17	Interviews as Topic/	55,343
18	*Needs Assessment/	8221
19	exp *Quality Assurance, Health Care/og, st	29,186
20	Vital Statistics/	5050
21	Medical Errors/	15,457
22	*Registries/	22,443
23	Injury Severity Score/	14,406
24	*Hospitalization/sn	11,858
25	*Quality Improvement/	9657
26	Benchmarking/	12,274
27	*Quality Indicators, Health Care/	7910
28	or/16-27	223,545
29	exp *"Delivery of Health Care"/ or Delivery of Health Care/mt, st	591,826
30	exp *Health Services Accessibility/	54,186
31	*"Health Services Needs and Demand"/	21,052
32	*"outcome assessment (health care)"/	26,940
33	*"outcome and process assessment (health care)"/	9293
34	*patient outcome assessment/	1611
35	*"Process Assessment (Health Care)"/	2337
36	*Risk Assessment/mt	13,979
37	*Triage/ or Triage/og or (Triage/ and Health Services Research/)	6076
38	Program Evaluation/	57,581
39	exp *Disaster Planning/mt, og	4659
40	or/29-39	692,765
41	15 and 28 and 40	3417
42	*Trauma Centers/st	470
43	((trauma* or postrauma*) adj3 (access* or capacit* or evaluat* or assess* or tool or tools or interview* or survey* or qualit* improv*)).ti,kf.	2044
44	((emergency* or emergencies*) adj3 (access* or capacit* or evaluat* or assess* or qualit* improv* or indicator*)).ti,kf.	1927
45	((trauma* or postrauma* or emergency* or emergencies*) adj3 (access* or capacit* or benchmark* or evaluat* or assess* or triage* or rapid* or checklist* or check-list* or survey* or questionnair* or tool or tools or interview* or indicator* or (qualit* adj1 (assurance* or improv* or measure* or control*)))).ab. /freq = 3	558
46	(three-delay* or ((trauma* or postrauma* or emergency* or emergencies*) adj3 ((time* or length or duration*) adj1 (delay* or factor*)))).tw,kf.	273
47	((trauma* or postrauma* or emergency* or emergencies*) and ((verbal* or social*) adj2 autops*)).tw,kf.	48
48	15 and ((verbal* or social*) adj2 autops*).tw,kw.	36
49	or/41-48	8359
50	Animals/ not (Animals/ and Humans/)	4,469,363
51	((animal or animals or cat or cats or dog or dogs or feline or hamster* or mice or monkey or monkeys or mouse or murine or pig or pigs or piglet* or porcine or primate* or rabbit* or rats or rat or rodent* or sheep*) not (human* or patient*)).ti,kf.	1,993,676
52	49 not (50 or 51)	8266
53	from 52 keep 1-5000	5000
54	remove duplicates from 53	4992
55	from 50 keep 5001-8266	3266
56	remove duplicates from 55	3266
57	54 or 56	8258

## Abbreviations

HIC: High-income country
LMIC: Low- and middle-income country
